# 2023 Outstanding Contributions to ISCB Award: Shoba Ranganathan

**DOI:** 10.1093/bioinformatics/btad306

**Published:** 2023-06-30

**Authors:** Christiana N Fogg, Diane E Kovats, Martin Vingron

**Affiliations:** Kensington, MD, United States; International Society for Computational Biology (ISCB), 525K East Market Street, RM 330, Leesburg, VA, United States; International Society for Computational Biology (ISCB), 525K East Market Street, RM 330, Leesburg, VA, United States; Max-Planck-Institute for Molecular Genetics, Computational Molecular Biology, Ihnestr. 73, Berlin 14195 D, Germany

The Outstanding Contributions to ISCB Award recognizes an ISCB member annually for notable service contributions toward the betterment of ISCB through exemplary leadership, education, and service. The 2023 Outstanding Contributions to ISCB Award recipient is Shoba Ranganathan. She will be recognized with this award at the 2023 ISMB/ECCB conference in Lyon, France.


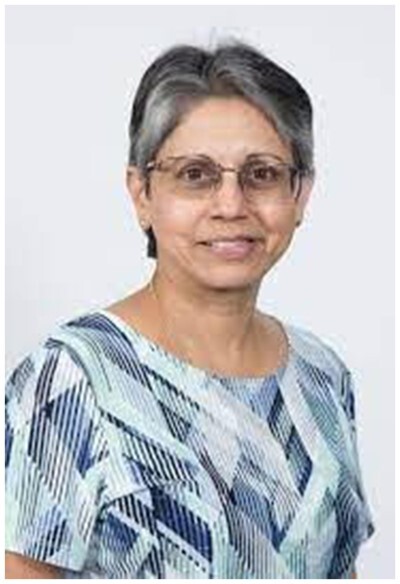
 *Prof. Shoba Ranganathan, FABACBS, Macquarie University*.

Shoba Ranganathan is a Professor of Bioinformatics at Macquarie University in Sydney, Australia. Ranganathan’s research interests include immunoinformatics, transcriptomics, and biodiversity informatics. She is a long-standing ISCB member and has served the greater bioinformatics community for over 20 years. Ranganathan was born and raised in India and received her PhD from the Indian Institute of Technology in Delhi. Her bioinformatics career has spanned the globe through academic and industry positions in India, France, the USA, Singapore, and Australia, which has given her a unique and valuable insight into bioinformatics research and education activities in diverse settings.

Shoba first became a member of ISCB in 1999 when she had a paper accepted at the Pacific Symposium of Biocomputing (PSB). It was there she met some of the pioneers of computational biology, including Russ Altman, Larry Hunter, Subramanian Subbiah, and Keith Dunker, among others. This led to her getting involved with the Asia-Pacific Bioinformatics Network (APBioNet), which was the first regional affiliate of ISCB. Shoba has held numerous leadership roles in APBioNet, including Vice-President (2000–4), President (2005–16), Advisory Board (since 2020), and Board of Directors (honorary) (2016–present). She has also built ISCB’s connections with other international scientific networks, including serving as a founding co-chair of CompMS [joint initiative of ISCB community of special interest (COSI), Human Proteome Organization, and the Metabolomics Society]. Shoba is a founding president (2003–5) of the Association for Medical and Bio Informatics Singapore (AMBIS), ISCB regional affiliate, and a founding member of GOBLET (Global Organization for Bioinformatics Learning, Education and Training) (2012–present) and hosted their annual meeting at the International Conference of Bioinformatics (InCoB) 2019. She has also been instrumental in facilitating the peer review of InCoB papers in *BMC Bioinformatics* (2006–present), followed by the addition of *BMC Genomics*, *BMC Medical Genomics*, *BMC Systems Biology*, and *BMC Cell and Molecular Biology*.

Ranganathan has directly served ISCB in various roles, including as a member of the ISCB Board of Directors (2002–6), on the Education Committee as Co-Chair (2003–4), Chair (2004–5), and current member, and as a Co-Chair of Affiliates Committee (2004–6). She campaigned for parallel sessions at ISMB, which was adopted from 2004, switching from the single session program until 2003. Her service has been pivotal to realizing ISCB’s role in promoting bioinformatics education. She recalled, “I moved to Singapore in August 2000, where I put forward a proposal for a Workshop on Education in Bioinformatics (WEB) for ISMB2001, organized by Søren Brunak. I kissed my bank account away signing a personal guarantee for the entire cost of this Special Interest Group meeting. It is gratifying to note that WEB is still on the agenda (as a COSI now), and fortunately, all SIG meetings are underwritten by the ISCB nowadays.”

Shoba’s service has been driven by a desire to better connect the global bioinformatics community. She still sees a “digital divide” among the bioinformatics communities in the Asia-Pacific, especially in under-resourced areas. Ranganathan has worked to connect these groups through activities with APBioNet, Bioinformatics Australia/ABACBS, ICSB, and other societies, which has been critical to improving bioinformatics education and supporting newly formed bioinformatics societies. Her work in this area has been pivotal in building bioinformatics education and infrastructure in Australia. Her work has been recognized with multiple awards, including the 2018 ABACBS Honorary Senior Fellowship, and as the first UNESCO Chair of Biodiversity Informatics in 2006.

Shoba remains deeply involved with the bioinformatics community, especially as she anticipates the global reach of bioinformatics to expand to applications including environmental and health research, synthetic biology and gene modifications, and artificial intelligence for biological knowledge integration and analysis. She is honored and grateful for her recognition with the 2023 Outstanding Contributions to ISCB Award and encourages junior scientists and trainees to seek out varied service opportunities to expand their knowledge and give back to their scientific community.

